# Filter bank temporally local multivariate synchronization index for SSVEP-based BCI

**DOI:** 10.1186/s12859-024-05838-y

**Published:** 2024-07-02

**Authors:** Tingting Xu, Zhuojie Ji, Xin Xu, Lei Wang

**Affiliations:** School of Communication and Information Engineering, Nanjing University of Posts and Telecommunications, Nanjing, 210003 Jiangsu China

**Keywords:** Brain–computer interface (BCI), Filter bank, Multivariate synchronization index (MSI), Steady-state visual evoked potential (SSVEP), Temporal local information

## Abstract

**Background:**

Multivariate synchronization index (MSI) has been successfully applied for frequency detection in steady state visual evoked potential (SSVEP) based brain–computer interface (BCI) systems. However, the standard MSI algorithm and its variants cannot simultaneously take full advantage of the time-local structure and the harmonic components in SSVEP signals, which are both crucial for frequency detection performance. To overcome the limitation, we propose a novel filter bank temporally local MSI (FBTMSI) algorithm to further improve SSVEP frequency detection accuracy. The method explicitly utilizes the temporal information of signal for covariance matrix estimation and employs filter bank decomposition to exploits SSVEP-related harmonic components.

**Results:**

We employed the cross-validation strategy on the public Benchmark dataset to optimize the parameters and evaluate the performance of the FBTMSI algorithm. Experimental results show that FBTMSI outperforms the standard MSI, temporally local MSI (TMSI) and filter bank driven MSI (FBMSI) algorithms across multiple experimental settings. In the case of data length of one second, the average accuracy of FBTMSI is 9.85% and 3.15% higher than that of the FBMSI and the TMSI, respectively.

**Conclusions:**

The promising results demonstrate the effectiveness of the FBTMSI algorithm for frequency recognition and show its potential in SSVEP-based BCI applications.

## Background

Brain–computer interface (BCI) is an emerging technology that provides a direct communication pathway between the brain and the external environment or devices [1, 2]. Steady-state visual evoked potential (SSVEP) measured by electroencephalography (EEG) is currently one of the most widely used control signals in BCI systems [3, 4]. SSVEP is defined as the brain response to a visual stimulus flickering at a fixed frequency. When users look at a target stimulus, the SSVEP signals can be observed at the same fundamental frequency as the stimulus, as well as the harmonics of the driving stimulus [5]. Due to high information transfer rate (ITR) [5, 6], little training [7, 8] and high reliability [9], SSVEP has been successfully applied in various BCI applications, including virtual keyboard systems [10, 11], brain-controlled wheelchairs [12], and robotic arm control systems [13].

For SSVEP-based BCI, a key problem is to identify the target frequency from the SSVEP signals. Currently, two mainstream SSVEP frequency detection methods are the canonical correlation analysis (CCA) [14] and multivariate synchronization index (MSI) [15]. The standard CCA method and its derivatives, such as filter bank CCA (FBCCA) [16], individual template based CCA (IT-CCA) [17], discriminative multiple CCA (DMCCA) [18], and filter bank temporally local CCA (FBTCCA) [19], calculate the canonical correlation coefficients between the multi-channel SSVEP signals and the reference signals. Different from CCA, the MSI algorithm estimates the synchronization index between the multi-channel signals and the reference signal, which has shown superior frequency recognition performance compared to CCA [15, 20]. Recently, several extended methods are proposed to improve the performance of standard MSI [20–23], including temporally local multivariate synchronization index (TMSI) [20], extension to MSI (EMSI) [21], inter- and intra-subject template-based MSI (IIST-MSI) [22], and filter bank MSI (FBMSI) [23]. The TMSI method improved MSI performance by utilizing the time-local information of SSVEP signal when modeling the covariance matrix [20]. The EMSI method combined the delayed version of EEG signal to improve the effectiveness of the CSP algorithm, resulting in higher recognition accuracy and ITR [21]. The IIST-MSI method employed the inter-subject similarity and variability through template transfer to enhance the robustness of SSVEP recognition [22]. The FBMSI method takes into account the information in EEG harmonic components and employed filter bank strategy to improve the frequency detection accuracy of SSVEP [23].

Although standard MSI and its variants have been successfully applied in various SSVEP-BCI systems, they are limited by not simultaneously utilizing the time-local structure and the harmonic components in SSVEP signals, both of which are crucial for frequency detection performance. On the one hand, the EEG signal is non-stationery and changes slowly over time [24]. The temporally local variances of signal have been proven to provide important information for capturing brain activity features in previous EEG studies [19, 20]. On the other hand, through multi-band decomposition strategy, the harmonic components in SSVEP signal have also shown its effectiveness in decoding EEG patterns in various BCI applications [19, 23].

In this study, we propose a novel FBTMSI algorithm for SSVEP frequency detection. Compared with the standard MSI algorithm, the proposed FBTMSI algorithm adds multiple frequency subbands containing different harmonics for analysis, which takes full advantage of the harmonic components in SSVEP. It also integrates the temporal structure of signals into the covariance matrix of the standard MSI, which further improves the identification performance. More specifically, multiple filter banks are designed to separate the SSVEP signal into several sub-bands in specific frequency ranges to improve the frequency resolution. The sub-band signals are then processed in the time domain for TMSI analysis, which captures the temporal local synchronization of SSVEP signals. Finally, the temporal local MSI of each sub-band is combined with specific weights to obtain a synchronization index that reflect the correlation between the original EEG signal and the reference signal, based on which the target frequency is determined. Following the cross validation scheme, we conduct extensive experiments on the public Benchmark dataset [25] to optimize the parameters and evaluate the performance of the proposed FBTMSI algorithm. Experimental results indicate that FBTMSI has higher recognition accuracy and ITR across multiple experimental settings, when compared with standard MSI [15], as well as the state-of-the-art TMSI [20] and FBMSI [23] algorithms.

The rest of this paper is organized as follows. In Section “Methods”, we present a detailed description of the proposed FBTMSI algorithm. Section "SSVEP dataset and performance evaluation” describes the SSVEP datasets and the performance evaluation procedure. Section “Parameter optimization and results analysis” discusses parameter optimization and analyzes the results of the FBTMSI algorithm. Section “Discussions and conclusions” presents the discussions and conclusions of this work.

## Methods

### The standard MSI method

The standard MSI method estimates the synchronization between the EEG signal and the reference signal as a potential indicator to identify the stimulus frequency. Since the SSVEP signal spectrum not only shows maximum amplitude at the fundamental frequency of the stimulus, but also local peaks at the harmonics, the harmonic components of the stimulus signal are incorporated in the reference signal.

Let $X\in {R}^{{N}_{c}\times M}$ denotes the multichannel EEG signal. ${N}_{c}$ represents the number of channels and $M$ is the number of time samples. The reference signal $Y\in {R}^{2{N}_{h}\times M}$ is defined as:

1 $$Y={Y}_{n}=\left[\begin{array}{c}sin\left(2\pi {f}_{n}t\right)\\ cos\left(2\pi {f}_{n}t\right)\\ \vdots \\ sin\left(2\pi {N}_{h}{f}_{n}t\right)\\ cos\left(2\pi {N}_{h}{f}_{n}t\right)\end{array}\right],t=\frac{1}{{F}_{S}},\frac{2}{{F}_{S}},\cdots ,\frac{M}{{F}_{S}}$$

where ${N}_{h}$ is the number of harmonics, ${f}_{n}$ represents the $n$ th stimulus frequency, and ${F}_{s}$ represents the sampling frequency. In this study, there are 40 stimulus frequencies, ${f}_{n}\in \left\{{f}_{1},{f}_{2},\ldots ,{f}_{40}\right\}$. Without loss of generality, the EEG signal $X$ and the reference signal $Y$ need to be normalized to have zero mean and unit variance. The joint covariance matrix of $X$ and $Y$ can be calculated as:

2 $$C=\left[\begin{array}{c}{C}_{11}=\frac{1}{M}X{X}^{T}{C}_{12}=\frac{1}{M}X{Y}^{T}\\ {C}_{21}=\frac{1}{M}Y{X}^{T}{C}_{22}=\frac{1}{M}Y{Y}^{T}\end{array}\right]$$

To reduce the impact of the autocorrelation of $X$ and $Y$ on subsequent synchronization calculations, the following linear transformation is applied:

3 $$Q=\left[\begin{array}{cc}{C}_{11}^{-\frac{1}{2}}& 0\\ 0& {C}_{22}^{-\frac{1}{2}}\end{array}\right]$$

Then, the new joint correlation matrix can be described as:

4 $$R=QC{Q}^{T}=\left(\begin{array}{cc}{\text{I}}_{{N}_{c}}& {C}_{11}^{-\frac{1}{2}}{C}_{12}{C}_{22}^{-\frac{1}{2}}\\ {C}_{22}^{-\frac{1}{2}}{C}_{21}{C}_{11}^{-\frac{1}{2}}& {\text{I}}_{2{N}_{h}}\end{array}\right)$$

where ${\text{I}}_{{N}_{c}}$ and ${\text{I}}_{2{N}_{h}}$ are identity matrices.

${\lambda }_{1},{\lambda }_{2},\ldots ,{\lambda }_{p}$ are the eigenvalues of the matrix $R$. The normalized eigenvalue ${\lambda }_{i}^{{{\prime}}}$ is calculated as follows:

5 $${\lambda }_{i}^{{{\prime}}}=\frac{{\lambda }_{i}}{\sum_{i=1}^{P}{\lambda }_{i} }=\frac{{\lambda }_{i}}{tr\left(R\right)}$$

Let $P={N}_{c}+2{N}_{h}$. The synchronization index, i.e., the normalized entropy between two multi-channel signals can be obtained as:

6 $$S=1+\frac{\sum_{i=1}^{P}{\lambda }_{i}^{{{\prime}}}\text{log}\left({\lambda }_{i}^{{{\prime}}}\right)}{\text{log}\left(P\right)}$$

According to (6), we can calculate the multivariate synchronization index ${S}_{n}$ between the EEG signal $X$ and the different reference frequency signals ${Y}_{n}$. Finally, the target frequency ${f}_{t}$ can be determined as the reference frequency corresponding to the maximum value of ${S}_{n}$.

7 $${f}_{t}=\underset{n}{arg\text{max}} {S}_{n}, n=\text{1,2},\cdots ,40$$

### Temporally local MSI

In the standard MSI method, the mutual synchronization of multichannel EEG signals is evaluated by calculating the eigenvalues and eigenvectors of the covariance matrix, as well as the normalized entropy, which has been shown to be an effective method for frequency identification in SSVEP-BCI systems. However, the standard MSI method ignores the temporal local information of EEG signals. To address this problem, the TMSI algorithm was proposed which has shown superior frequency recognition accuracy compared with standard MSI [20].

The TMSI algorithm defines the adjacency matrix $W\in {R}^{M\times M}$ and multiple signals $Z=\left[{z}_{1},{z}_{2},\ldots ,{z}_{M}\right]\in {R}^{{N}_{c}\times M}$, respectively. ${N}_{c}$ denotes the number of variables or channels while $M$ denotes the number of time samples. Then, the temporal local covariance matrix is expressed as follows:

8 $${C}^{{{\prime}}}=\frac{1}{2M}\sum_{i=1}^{M}\sum_{j=1}^{M}{W}_{i,j}\left({z}_{i}-{z}_{j}\right){\left({z}_{i}-{z}_{j}\right)}^{T}$$

Equation (8) can be transformed into

9 $$\begin{aligned}{C}^{{{\prime}}}&=\frac{1}{M}\sum_{i=1}^{M} {z}_{i}{z}_{i}^{T}\sum_{j=1}^{M} {W}_{i,j}-\sum_{i=1}^{M} \sum_{j=1}^{M} {W}_{i,j}{z}_{i}{z}_{j}^{T}\\ &=\frac{1}{M}\left(Z(D-W){Z}^{T}\right)\\ &=\frac{1}{M}ZL{Z}^{T}\end{aligned}$$

where $D$ is the diagonal matrix, for $i=1,2,\ldots ,M$,${D}_{i,i}=\sum_{j=1}^{M} {W}_{i,j}$. $L$ is the Laplacian matrix and$L=D-W$. The adjacency matrix $W$ can be generated by in a variety of ways. In this paper, it is determined by Tukey’s tricube weighting function [26]:

10 $${W}_{i,j}=\left\{\begin{array}{c}{\left(1-{\left|\frac{i-j}{\tau }\right|}^{3}\right)}^{3},\left|\frac{i-j}{\tau }\right|<1\\ 0, else\end{array}\right.$$

Based on (2) and (9), a new temporal local covariance matrix can be calculated as

11 $${C}^{{{\prime}}}=\left[\begin{array}{cc}\frac{1}{M}XL{X}^{T}& \frac{1}{M}XL{Y}^{T}\\ \frac{1}{M}YL{X}^{T}& \frac{1}{M}YL{Y}^{T}\end{array}\right]$$

When ${C}{\prime}$ is determined, we can use (2)–(6) to calculate the synchronization index, and use (7) to implement frequency identification.

### FBTMSI

**Fig. 1:**
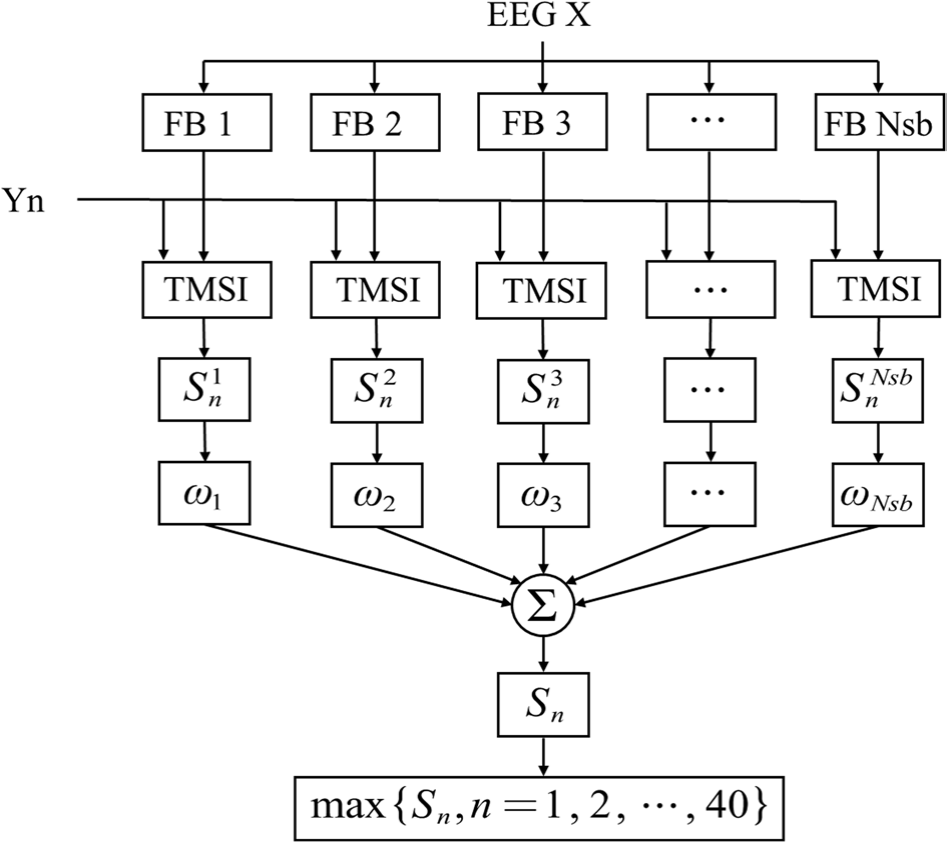
Flow chart of FBTMSI method

TMSI benefits from exploiting the temporal local structure of EEG signals. However, it ignores the harmonic components of SSVEP, which have been shown to be informative for frequency identification [16, 27]. Filter bank approach has been successfully applied in previous studies to extract the information contained in harmonics [16, 19, 23]. Based on these ideas, we propose a novel FBTMSI method that takes into account both the temporal local structure and the harmonic components in SSVEP signal to improve frequency detection performance. The flowchart of FBTMSI algorithm is shown in Fig. 1.

First, filter banks are applied on the original EEG signal to decompose the signal into multiple frequency subbands. According to the filter bank design method in [16], a zero-phase Chebyshev type I IIR filter is employed to extract each subband component ($F{B}_{l},l=1,2,\cdots ,{N}_{sb}$, where $l$ denotes the subband index) from the original EEG signal $X$. After the filter bank analysis, the temporally local multivariate synchronization index between each subband component and the reference signal corresponding to each stimulus frequency is then calculated respectively.

**Fig. 2:**
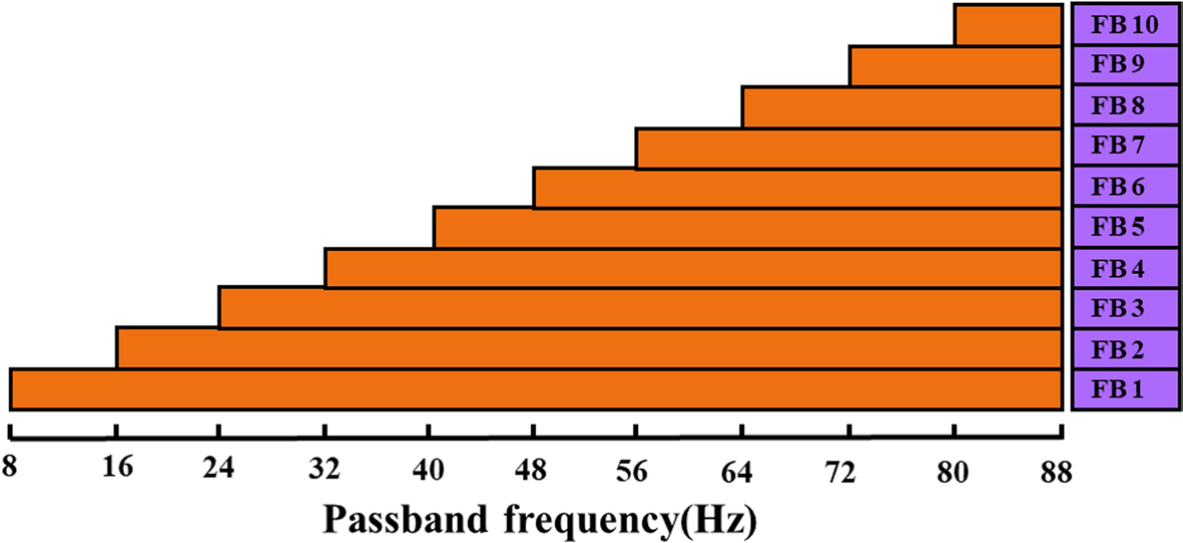
Frequency range corresponding to each subband of the FBTMSI algorithm

In this study, the bandwidth of the stimulation frequency (8–15.8 Hz) was 8 Hz. According to [16], the fundamental and harmonic components exhibit high signal-to-noise ratio (SNR) in the upper-frequency band from the stimulation frequency to about 90 Hz. Therefore, we chose the frequency range within [8 Hz, 88 Hz] (10 times the bandwidth of the stimulus frequency) as the filter bank. The frequency range is divided into ${N}_{sb}$ subbands, each with a frequency range of$\left[\text{8,88}\right]\text{Hz}, \left[\text{16,88}\right]\text{Hz}, \left[l,88\right]\text{Hz }\ldots [{N}_{sb}\times 8,88\text{Hz}]$, as shown in Fig. 2. Then, TMSI is performed on each subband signal and the reference signal${Y}_{n}$, to calculate the corresponding TMSI index${S}_{n}^{l}$. Since the SNR of the harmonic component of the SSVEP signal decreases with increasing frequency [23], we performed a weighted sum of ${S}_{n}^{l}$ corresponding to each subband, and the weighting factor ${\omega }_{l}$ corresponding to each ${S}_{n}^{l}$ is shown in (12):

12 $${\omega }_{l}={l}^{-a}+b$$

where $a$ and $b$ are constants. Then, the weighted sum ${\text{S}}_{n}$ of each subband’s TMSI is calculated as an indicator of FBTMSI, as shown in (13). Finally, the same (7) is used to implement frequency identification.

13 $${S}_{n}=\left[{S}_{n}^{1},{S}_{n}^{2},\cdots ,{S}_{n}^{Nsb}\right]\left[\begin{array}{c}{\omega }_{1}\\ {\omega }_{2}\\ \vdots \\ {\omega }_{Nsb}\end{array}\right]$$

## SSVEP dataset and performance evaluation

### SSVEP dataset

**Fig. 3:**
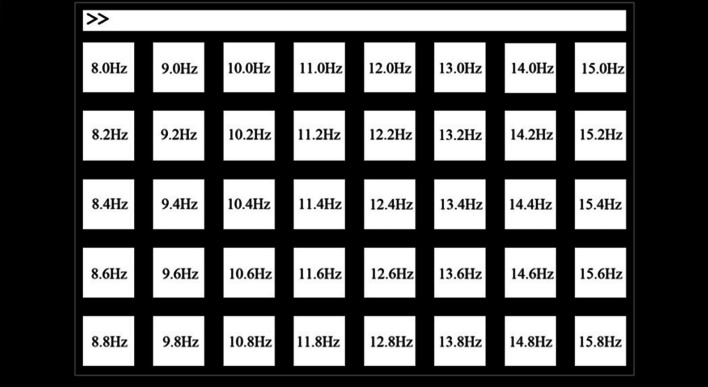
Stimulation frequencies corresponding to the 40 targets [25]

**Fig. 4:**
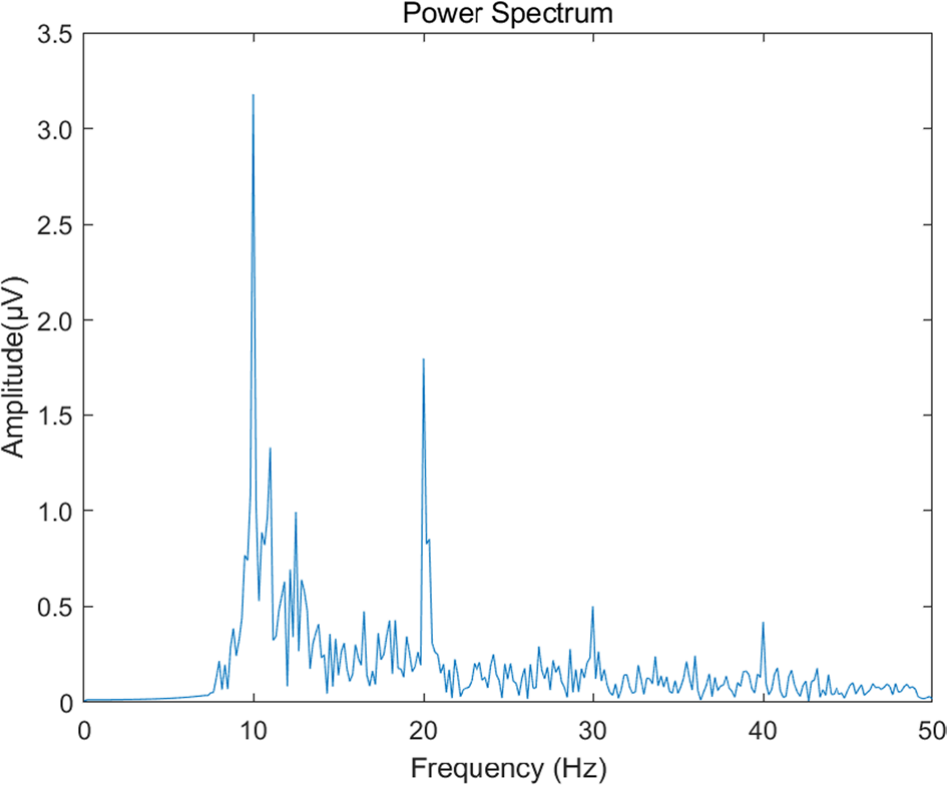
EEG spectrum of SSVEP triggered by 10 Hz visual stimulation frequency

The EEG data used in this study are from the public SSVEP Benchmark dataset of 35 subjects [25]. The experiment consisted of 6 blocks, each containing 40 frequency trials, and each trial used 40 characters presented in random order corresponding to different frequencies (8–15.8 Hz with an interval of 0.2 Hz) of flicker. The schematic is detailed in Fig. 3. Experiments were recorded using the Neuroscan Synamps2 EEG system with a sampling rate of 1000 Hz, and the signals were pre-processed with a 0.15–200 Hz bandpass filter and a 50 Hz trap filter. The recorded SSVEP signals were down-sampled to 250 Hz to improve data processing efficiency. Each trial contained 1500 time points lasting a total of 6 s, with the first 0.5 s used for cue presentation, 5 s for stimulus presentation, and the remaining 0.5 s for blanking and rest. Figure 4 shows an example of EEG spectrum of SSVEP triggered by 10 Hz visual stimulation frequency.

### Performance evaluation

A five-fold cross-validation procedure was employed to optimize the algorithm parameters and evaluate the performance of the proposed FBTMSI algorithm. For each fold, all data blocks of 7 subjects were left out for testing while the rest data blocks of 28 subjects were used for parameter optimization. The procedure was repeated 5 times until all subjects were tested. We conducted cross-validation in different time windows. This procedure takes into account the variability in brain signal among different individuals, which grantees the generalization ability of the proposed algorithm.

To evaluate the performance of the proposed FBTMSI algorithm, we compare the frequency detection accuracy and ITR of FBTMSI under different time windows (TW) with the standard MSI [15], as well as the state-of-the-art TMSI [20] and FBMSI [23] algorithms. Detection accuracy was defined as$\frac{{N}_{correct\, identification\, trials}}{{N}_{total\, number\, of\, trials}}$. The time windows were chosen to be [0.5 s: 0.5 s: 3 s]. The frequency band range was consistently set to [8, 88] Hz in different methods.

**Fig. 5:**
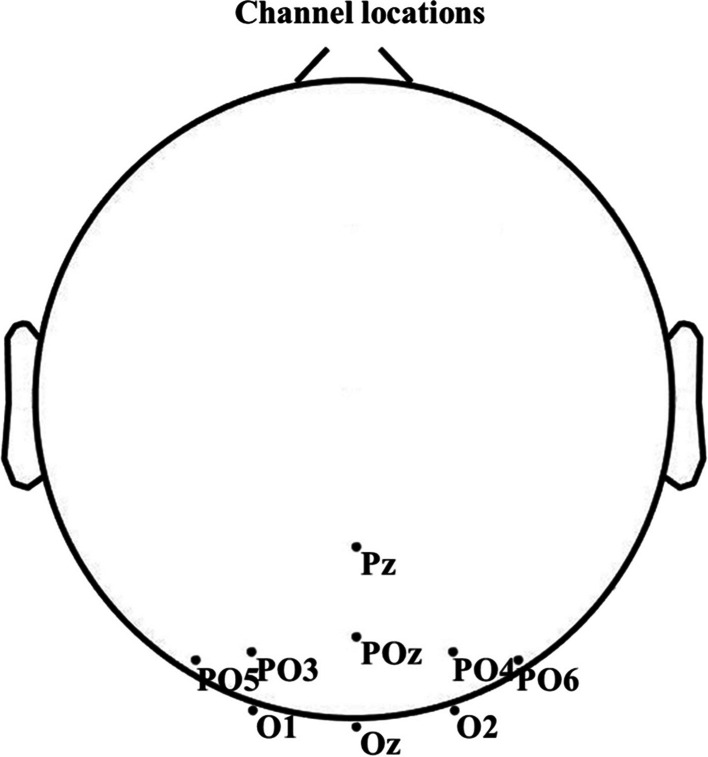
Channel location

In this study, nine EEG electrodes from the parietal and occipital brain regions were chosen for SSVEP analysis, as they contain the most informative SSVEP components [28]. The locations of these electrodes are shown in Fig. 5. In vision systems, the visual delay process needs to be considered [29]. Based on the recommendations of previous studies, a time delay of 140 ms was chosen for SSVEP analysis [16]. For each time window in the ITR calculation, a time interval of 0.5 s and a time delay of 140 ms were added for subjects to shift their vision.

## Parameter optimization and results analysis

### Performance evaluation

#### Optimization of harmonic number ${{\varvec{N}}}_{{\varvec{h}}}$

**Fig. 6:**
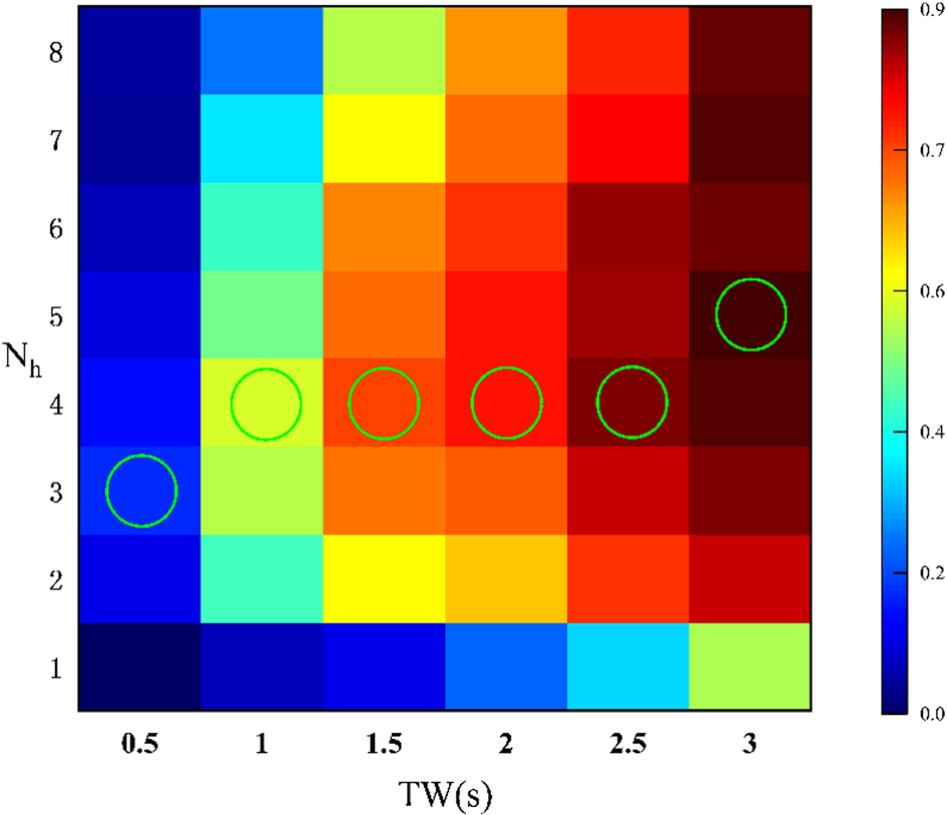
Relationship between recognition accuracy and the number of harmonics of the reference signal

Previous studies have shown that the number of harmonics ${N}_{h}$ in the reference signal has a considerable influence on recognition accuracy. For example, satisfactory results were achieved when ${N}_{h}$ = 2 in [20] and ${N}_{h}$ = 3 in [21], respectively. As shown in Fig. 6, when TW = 0.5 s, the highest recognition accuracy of the FBTMSI algorithm is achieved at ${N}_{h}$ = 3. When TW = 1 s, 1.5 s, 2 s and 2.5 s, the highest recognition accuracy is achieved at ${N}_{h}$ = 4. When TW = 3 s, the highest recognition accuracy is achieved at ${N}_{h}$ = 5.

It can be seen from Fig. 6 that the recognition accuracy of FBTMSI gradually improves as the length of the time window increases. It can be inferred that when the length of SSVEP signal is too short, the high-frequency harmonic peaks are not obvious and the corresponding SNR is low. As the SSVEP signal length increases, the SNR of the higher harmonics reaches a certain level and increasing the number of reference signal harmonics will improve the recognition accuracy. Since this study mainly investigates the performance of the FBTMSI at 0 to 3 s signal length, ${N}_{h}$ = 4 is the most suitable reference signal harmonic number for the FBTMSI algorithm.

#### Optimization of the number of filter banks ${{\varvec{N}}}_{{\varvec{s}}{\varvec{b}}}$

**Fig. 7:**
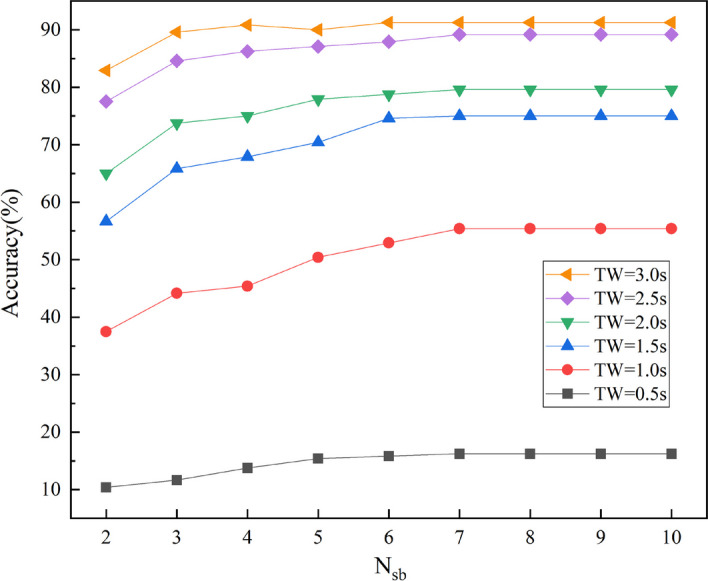
Recognition accuracy at different number of sub-bands

Figure 7 shows the effect of the number of filter banks on the recognition accuracy under different time windows (${N}_{sb}$ range set to [2, 10], FBTMSI is equivalent to TMSI when ${N}_{sb}$= 1). Overall, the recognition accuracy increases with the increase of the number of filter banks, but meanwhile, the computational cost increases, which affects the transmission efficiency. As can be seen from Fig. 7, for TW = 0.5 s to 3 s, the FBTMSI algorithm achieves the highest recognition accuracy when ${N}_{sb}$ = 7. To ensure that the SSVEP signals of different lengths are identified with high accuracy without sacrificing transmission efficiency, ${N}_{sb}$ = 7 was chosen as the filter bank number for the FBTMSI algorithm.

#### Optimization of temporal local parameters ${\varvec{\tau}}$ and weighting formula ${\varvec{\omega}}$

**Fig. 8:**
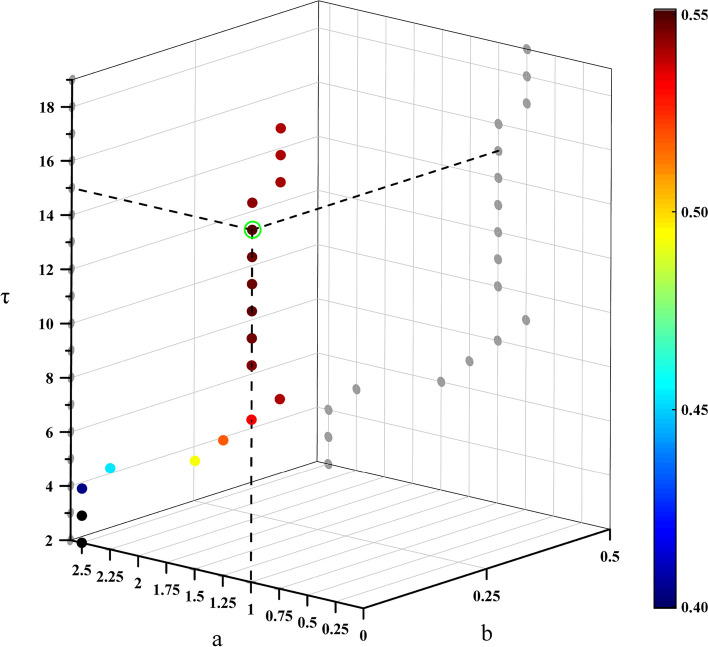
Optimization of the 3D grid search for parameters $\tau$, $a$, $b$

**Fig. 9:**
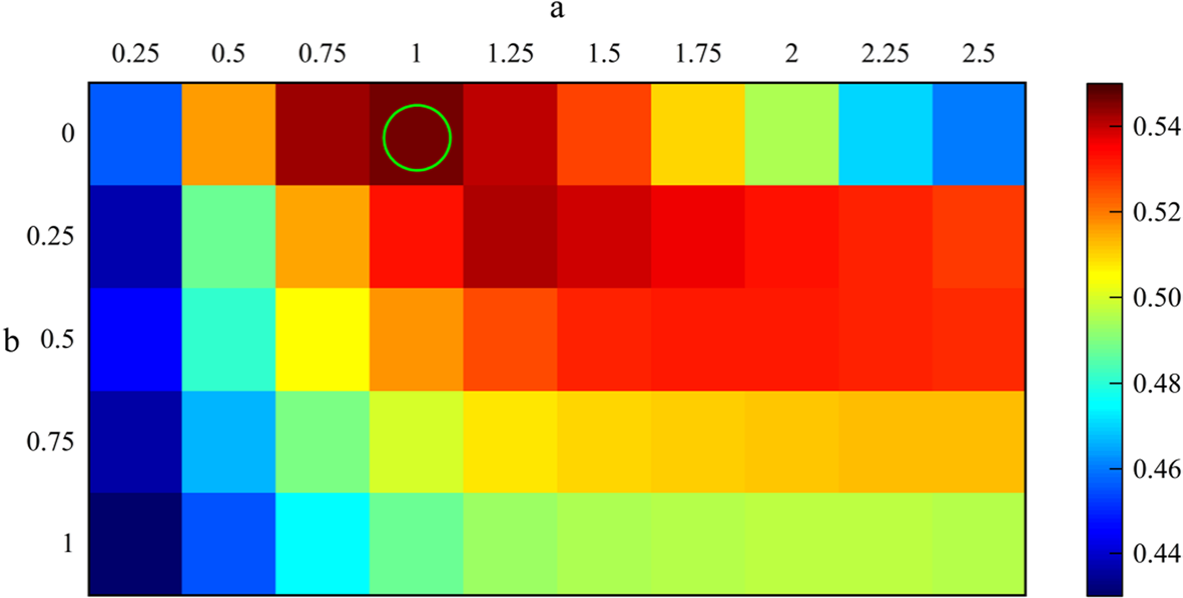
Optimization of the 2D grid search for parameters $a$ and $b$ at $\tau$=15

In the FBTMSI algorithm, three parameters need to be determined, namely the temporal local parameter $\tau$, $a$ and $b$ in the correlation coefficient weight $\omega$ corresponding to each subband. This is because the frequency range contained in each sub-band varies and the weight $\omega$ corresponding to TMSI in each subband changes. In this study, $\tau$, $a$, $b$ are determined using a 3D grid search method and screened in the ranges of [2:1:19], [0.25:0.25:2.5], and [0:0.25:1], respectively. It can be seen from Figs. 8 and 9 that the corresponding FBTMSI algorithm achieves the highest average recognition accuracy when ($\tau$, $a$, $b$) = (15,1,0).

### Results analysis

**Fig. 10:**
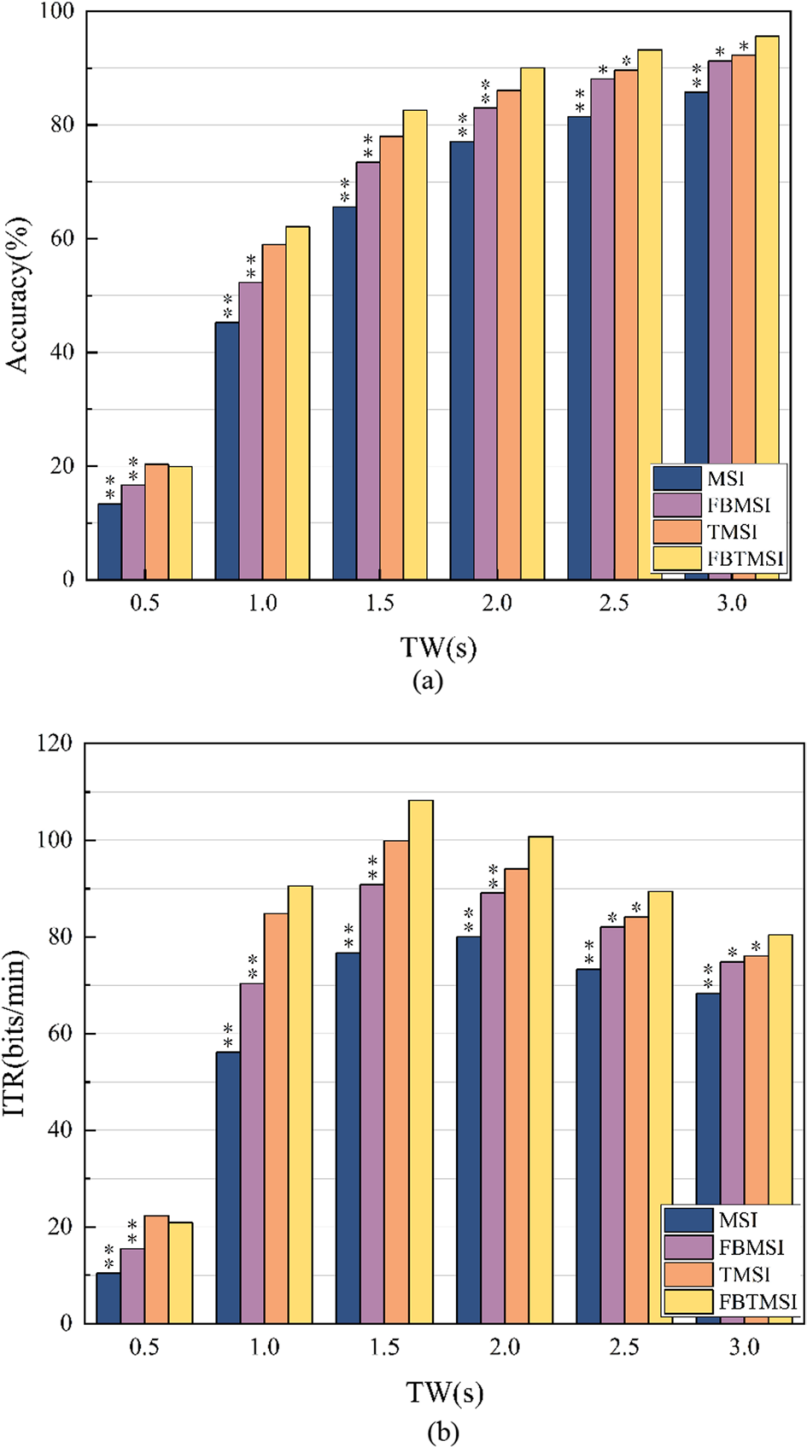
Average accuracy (**a**) and ITR (**b**) of 35 subjects at different TWs. Asterisks indicate significant differences between FBTMSI and the other method (*p < 0:05, **p < 0:01)

Tables 1 and 2 show the average recognition accuracy and ITR of all subjects under different TW (0.5–3 s, step size 0.5 s) of the proposed FBTMSI algorithm, as well as the standard MSI, TMSI and FBMSI algorithms for comparison. The performance of FBTMSI were higher than those of FBMSI and MSI at all six TWs, and higher than those of TMSI at TW from 1 to 3 s. In Fig. 10, we show the statistical analysis results using paired T-test in SPSS. The performance of FBTMSI of the 35 subjects was significantly higher than that of FBMSI and MSI in all six time windows and was significantly higher than that of TMSI at TW = 2.5 s and TW = 3.0 s.

**Table 1:** Average recognition accuracy (%) of the MSI, FBMSI, TMSI and FBTMSI algorithms at different TWs

TW	Method			
	MSI	FBMSI	TMSI	FBTMSI
0.5s	13.36 ± 5.97	16.67 ± 7.22	20.32 ± 9.60	19.93 ± 7.76
1.0s	45.25 ± 18.21	52.29 ± 20.09	58.99 ± 21.46	62.14 ± 18.94
1.5s	65.62 ± 22.33	73.44 ± 20.85	78.01 ± 20.11	82.62 ± 16.85
2.0s	77.04 ± 22.08	82.98 ± 18.21	86.07 ± 15.80	90.07 ± 12.54
2.5s	81.42 ± 20.45	88.10 ± 14.42	89.64 ± 12.30	93.21 ± 8.81
3.0s	85.75 ± 17.41	91.26 ± 11.95	92.24 ± 10.38	95.58 ± 6.02

**Table 2:** Average ITR (bits/min) of the MSI, FBMSI, TMSI and FBTMSI algorithms at different TWs

TW	Method			
	MSI	FBMSI	TMSI	FBTMSI
0.5s	10.41 ± 8.07	15.51 ± 11.38	22.30 ± 17.01	20.90 ± 13.32
1.0s	56.18 ± 32.51	70.34 ± 38.66	84.86 ± 43.89	90.56 ± 39.14
1.5s	76.68 ± 36.09	90.83 ± 36.91	99.83 ± 37.09	108.22 ± 31.98
2.0s	80.01 ± 32.52	89.14 ± 29.06	94.11 ± 26.36	100.77 ± 21.75
2.5s	73.35 ± 27.03	82.06 ± 20.74	84.12 ± 18.54	89.38 ± 13.96
3.0s	68.33 ± 20.92	74.88 ± 15.51	76.04 ± 14.04	80.42 ± 8.95

**Fig. 11:**
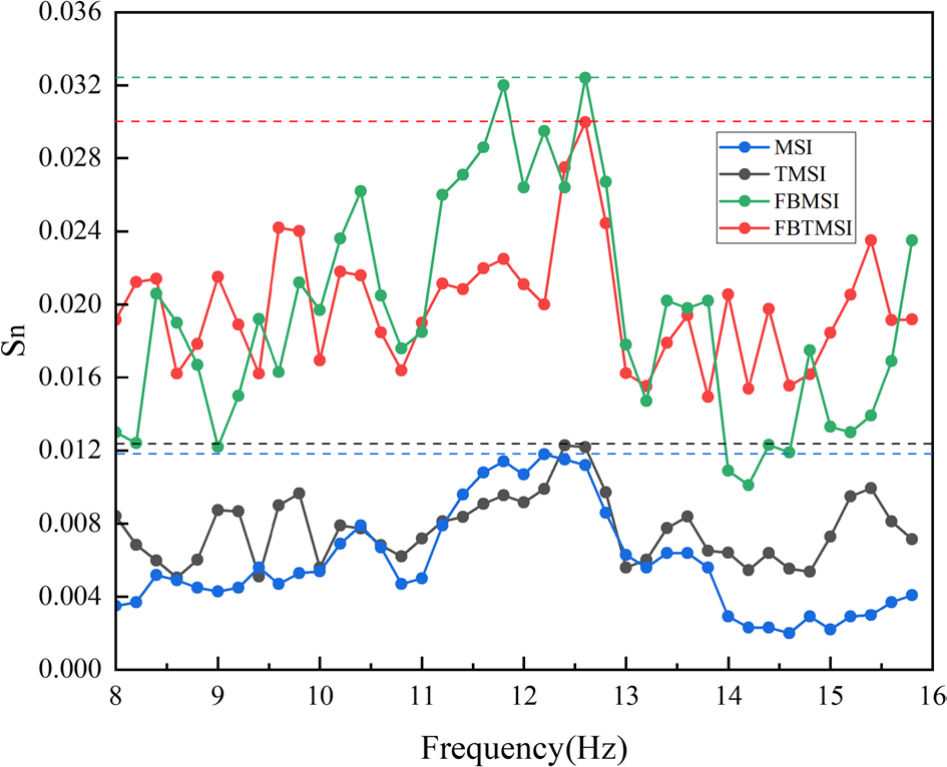
Frequency detection results of the MSI, FBMSI, TMSI and FBTMSI at visual stimulation frequency of 12.6 Hz

In Fig. 11, we show an example of frequency detection results at visual stimulation frequency of 12.6 Hz. FBTMSI and FBMSI achieve the highest ${S}_{n}$ value at 12.6 Hz and correctly identify the target frequency while TMSI reaches its peak at a different frequency of 12.4 Hz and MSI reaches its peak at 12.2 Hz, causing frequency recognition errors. This shows that by utilizing multiple filter banks, FBTMSI captures the frequency information more accurately compared to TMSI. The averaging of the filter banks also reduces the effect of noise, leading to more stable and robust results. Table 3 shows the frequency detection results at 40 visual stimulation frequencies in one block. Overall, the performance of FBTMSI is better than FBMSI and TMSI, and significantly better than standard MSI algorithm.

**Table 3:** Frequency detection results of the MSI, FBMSI, TMSI and FBTMSI in one block

Target frequency	Method			
	MSI	TMSI	FBMSI	FBTMSI
8	10.8	10.8	10.8	**8**
9	**9**	**9**	**9**	**9**
10	**10**	**10**	**10**	**10**
11	**11**	**11**	**11**	**11**
12	11.2	**12**	**12**	**12**
13	**13**	**13**	**13**	**13**
14	10.8	**14**	10.8	**14**
15	13	13	**15**	**15**
8.2	**8.2**	**8.2**	**8.2**	**8.2**
9.2	**9.2**	**9.2**	11.4	**9.2**
10.2	10.4	11.4	**10.2**	**10.2**
11.2	11.4	**11.2**	**11.2**	**11.2**
12.2	11.4	**12.2**	**12.2**	**12.2**
13.2	**13.2**	**13.2**	**13.2**	**13.2**
14.2	**14.2**	**14.2**	**14.2**	**14.2**
15.2	11	**15.2**	**15.2**	**15.2**
8.4	**8.4**	**8.4**	**8.4**	**8.4**
9.4	**9.4**	**9.4**	**9.4**	**9.4**
10.4	**10.4**	**10.4**	**10.4**	**10.4**
11.4	**11.4**	**11.4**	**11.4**	**11.4**
12.4	12.2	12.2	12.2	12.2
13.4	10.4	10.4	10.4	**13.4**
14.4	13.4	14.2	11.6	14.2
15.4	11.2	14.2	11.4	14.2
8.6	**8.6**	**8.6**	**8.6**	**8.6**
9.6	**9.6**	**9.6**	**9.6**	**9.6**
10.6	**10.6**	12.4	10.4	10.4
11.6	**11.6**	**11.6**	**11.6**	**11.6**
12.6	12.2	12.4	**12.6**	**12.6**
13.6	10.2	**13.6**	**13.6**	**13.6**
14.6	13.8	14.8	14.8	14.8
15.6	13.2	**15.6**	**15.6**	**15.6**
8.8	12.8	**8.8**	**8.8**	**8.8**
9.8	**9.8**	**9.8**	**9.8**	**9.8**
10.8	**10.8**	**10.8**	**10.8**	**10.8**
11.8	11.6	**11.8**	11.6	11.6
12.8	**12.8**	**12.8**	**12.8**	**12.8**
13.8	11.4	**13.8**	**13.8**	**13.8**
14.8	10.4	**14.8**	10.4	**14.8**
15.8	10.6	**15.8**	**15.8**	**15.8**
Correct number	19	30	29	34

The bold font denotes the correct frequency detection

**Fig. 12:**
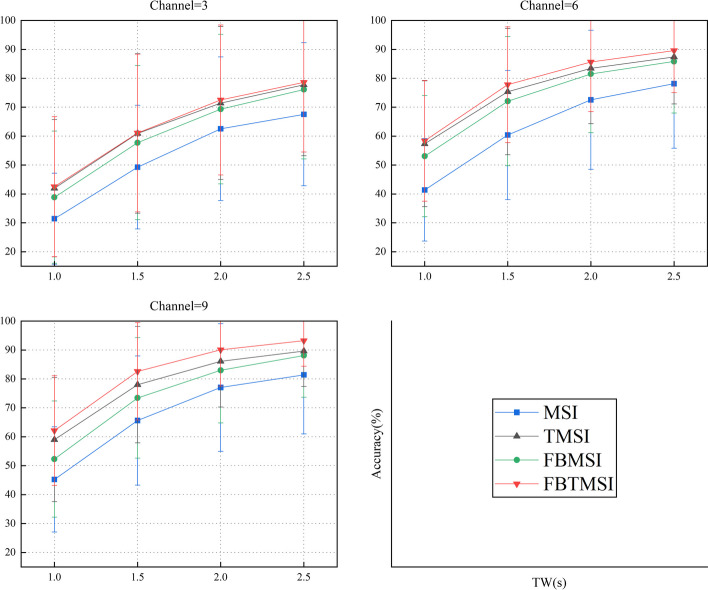
Average recognition accuracy of the MSI, TMSI, FBMSI and FBTMSI methods under different channels. Channel = 3, 6, 9 and time window lengths (TWs) from 1 s to 2.5 s. Channel = 3: Oz, O1 and O2; Channel = 6: PO3, PO4, Poz, Oz, O1 and O2; Channel = 9: PO3, PO4, PO5, PO6, Poz, Oz, O1 and O2. The errorbar denotes the standard deviation of accuracy on all the 35 subjects

With other conditions being equal, we compared the effect of channel numbers on frequency identification accuracy of the four methods, as shown in Fig. 12. These channels all come from the parietal and occipital lobes of the brain [28]. In Fig. 12, when channel = 3, the recognition accuracy of FBTMSI, FBMSI, and TMSI is significantly higher than that of MSI, but the improvement of FBTMSI is not obvious compared to FBMSI and TMSI. As the number of channels increases, the recognition accuracy of FBTMSI improves more significantly than that of FBMSI and TMSI.

## Discussions and conclusions

### Discussions

#### Design of filter banks

In order to study the influence of different filter banks on the accuracy of FBTMSI, three other sub-band design methods (a-c) were compared with the filter banks used in this paper (d).

**Fig. 13:**
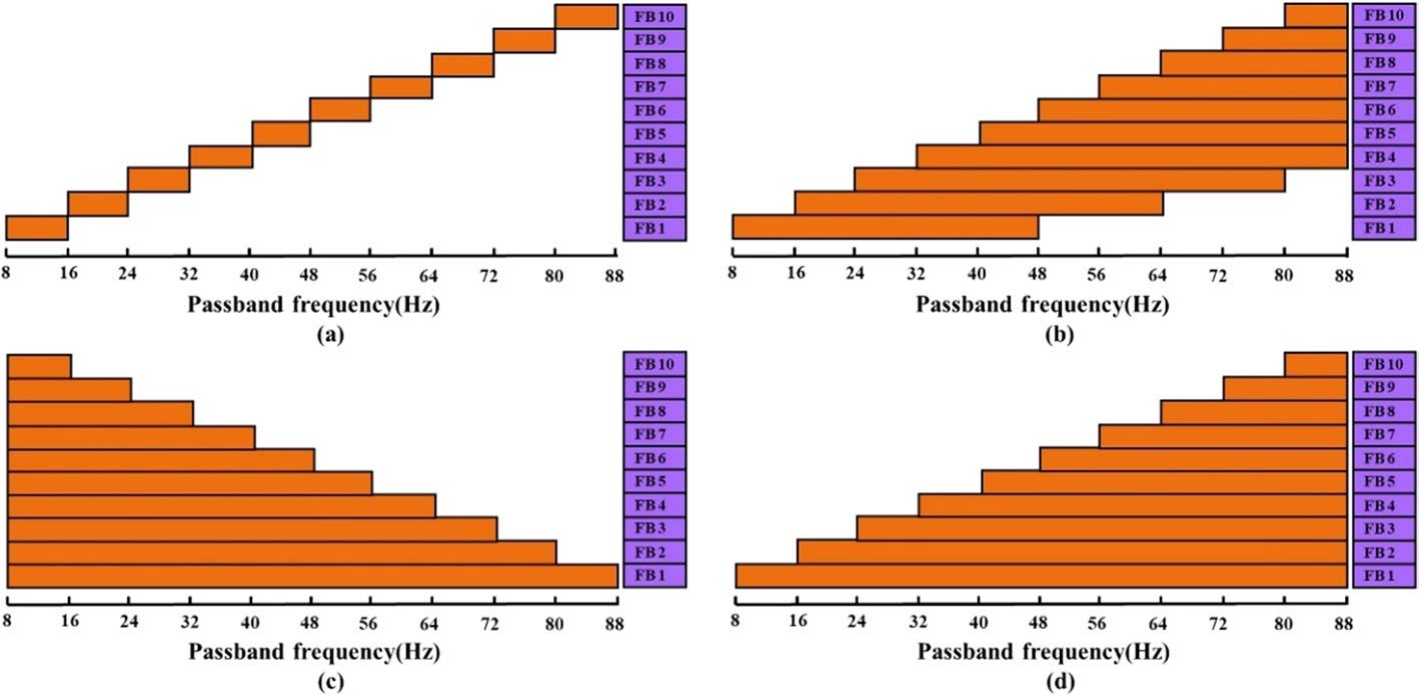
Four filter bank design methods for the FBTMSI algorithm

(a) The full frequency band of the SSVEP component is divided into sub-bands with equal intervals of bandwidth (frequency range of the stimulated signal), Fig. 13. (a) Filter range:$[8\times l, 8\times l+8]$ Hz.

(b) Each subband contains three times the bandwidth of the stimulated signal, Fig. 13. (b) Filter range:$[8\times l, min(16\times l+32, 88)]$ Hz.

(c) The lower cutoff frequency of each subband is 8 Hz, Fig. 13. (c) Filter range:$[8, 8\times (12-l)]$ Hz.

(d) The upper cutoff frequency of each subband is 88 Hz, Fig. 13. (d) Filter range: $[8\times l, 88]$ Hz.

**Fig. 14:**
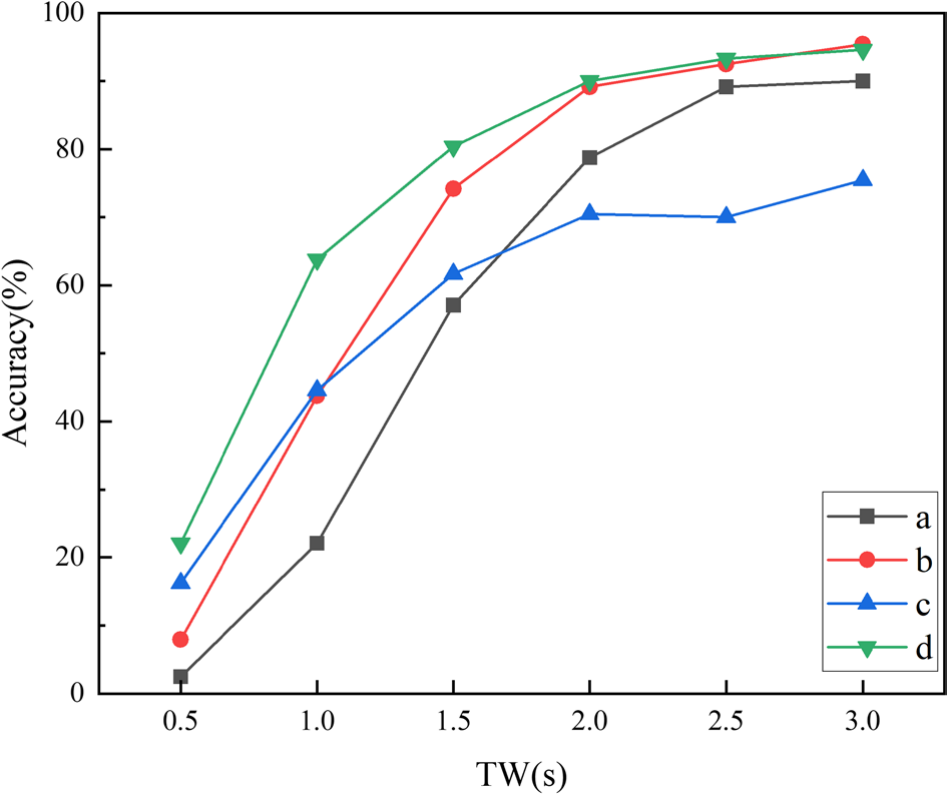
Recognition accuracy of the FBTMSI algorithm at different TWs under four filter subband design approaches

The SSVEP recognition accuracies of the four subband methods at different TWs are given in Fig. 14. Method (d) used in this study outperforms the other three methods in terms of recognition accuracy. The paired t-test shows a significant difference between (a) and the other methods (*p* < 0.05), while the difference between (b) and (d) is not significant (*p* > 0.05). These results suggest that the recognition improves as the bandwidth of each sub-band increases.

Methods (c) and (d) have an equal number of harmonics but differ in sub-band bias: (c) toward lower harmonics, while (d) toward higher harmonics. There is a significant difference in recognition accuracy between these two methods (*p* < 0.001). The results indicate that high harmonics play a crucial role in SSVEP frequency identification. Furthermore, with an increase in the length of SSVEP signal, the SNR of high harmonics also increases, indicating a higher available value. Figure 14 shows that as the signal length increases, the advantage of method (d) becomes more pronounced. These findings confirm the previous inference regarding harmonic optimization.

#### Weighting formula for the subbands

The grid search method was employed to determine the three parameters $\tau$, $a$ and $b$ for FBTMSI. $\tau$ establishes the relationship between two data points in the neighboring time range in TMSI, $a$, $b$ determines the weighting factor $\omega$ for each sub-band after through the filter bank. In this paper, the power exponential formula in (12) was used to determine the weights for each subband components. The rationale is that the SNR of the SSVEP harmonics decreases as the frequency increases. SNR is an important quantifier in SSVEP studies [30]. The SNR of an SSVEP at frequency ${f}_{n}$ is defined as the ratio of the power of the SSVEP at frequency ${f}_{n}$ to the average power at the surrounding $m$ frequencies:

14 $$\text{SNR}\left({f}_{n}\right)=P\left({f}_{n}\right)/\left(\frac{1}{m}\sum_{\begin{array}{c}q=-\frac{m}{2}\\ q\ne 0\end{array}}^\frac{m}{2} P\left({f}_{n+q}\right)\right)$$

where $P({f}_{n})$ denotes the power of the SSVEP at frequency ${f}_{n}$. In this paper, $m$ = 10 and the interval between adjacent frequencies is $\Delta f=0.2$ Hz. As frequency increases, the SNR of harmonic components decreases, which affects the performance of frequency detection. Therefore, in the weight assignment formula, high-frequency harmonic components are given higher weights to low-frequency components.

#### Comparison with other methods

In recent years, a number of SSVEP frequency detection methods have been proposed, such as FBCCA [16], FBTCCA [19], TMSI [20], EMSI [21] and EBMSI [23].These methods are all based on reference signals without training, which greatly reduces the experimental and training costs while ensuring recognition performance. We compared the performance of the proposed FBTMSI method with the above methods. To ensure a fair comparison, we conducted cross-validation for all above methods based on the Benchmark dataset [25]. All data blocks of 35 subjects were intercepted for analysis at [0.64 2.64]s (stimulation started at 0.5 s), and the frequency band range was set to [8, 88] Hz. Table 4 shows the average recognition accuracy and ITR of all 35 subjects for different methods.

**Table 4:** Performance comparison of the proposed FBTMSI method with other methods at TW = 2 s

Methods	Accuracy (%)	ITR (bits/min)
FBCCA	84.77	92.01
FBTCCA	86.46	94.61
EMSI	83.83	90.95
TMSI	86.07	94.11
FBMSI	82.98	89.14
FBTMSI	90.07	100.77

As can be seen from Table 4, compared with methods based on no training reference signals proposed in recent years, the proposed FBTMSI method has improved both accuracy and ITR. This demonstrates the effectiveness of FBTMSI in SSVEP-based BCI.

### Conclusions

In this paper, we propose a novel FBTMSI algorithm to improve SSVEP frequency detection performance based on MSI, which takes the full advantage of the time-local structure and the harmonic components in SSVEP signals. To our knowledge, no related work has combined the temporal and spectral features of SSVEP signal in MSI, which are both crucial for frequency detection performance. The proposed FBTMSI method explicitly utilizes the temporal information of signal for estimating covariance matrix and employs filter bank decomposition to exploits SSVEP-related harmonic components. Following the cross validation procedure, FBTMSI showed superior performance regarding recognition accuracy and ITR compared with the standard MSI, as well as the state-of-the art TMSI and FBMSI algorithms. These promising results demonstrate the effectiveness of the FBTMSI algorithm for frequency recognition and show its potential in SSVEP-based BCI applications.

## Data Availability

The public SSVEP Benchmark dataset of 35 subjects are downloaded from Tsinghua BCI Lab (http://bci.med.tsinghua.edu.cn/download.html).
